# Acknowledgement to Reviewers of *Veterinary Sciences* in 2018

**DOI:** 10.3390/vetsci6010007

**Published:** 2019-01-11

**Authors:** Veterinary Sciences Editorial Office

**Affiliations:** MDPI, St. Alban-Anlage 66, 4052 Basel, Switzerland

Rigorous peer-review is the corner-stone of high-quality academic publishing. The editorial team greatly appreciates the reviewers who contributed their knowledge and expertise to the journal’s editorial process over the past 12 months. In 2018, a total of 98 papers were published in the journal, with a median time to first decision of 21 days and a median time to publication of 48.5 days. The editors would like to express their sincere gratitude to the following reviewers for their cooperation and dedication in 2018:
Abdel-Whab, SayedLutsenko, SvetlanaAdams, GMachovsky-Capuska, GabrielAder, MarilynMackenstedt, UteAdler, Gregory H.Madeira De Carvalho, Luis ManuelAlberdi, PilarMahony, Timothy JohnAlessandri, ClaudiaMahsoub, HassanAllavena, RachelMair, TimAllen, Lee R.Malinowski, Robert P.Alvarez, Carlos E.Mancianti, FrancescaAndré, Marcos RogérioMani, RinoshArechiga-Ceballos, NidiaManiscalco, LorellaArtemiou, ElpidaMari, LorenzoAzaizeh, HassanMartano, MarinaBangoura, BeritMartín Castillo, MargaritaBarker, StephenMaurelli, Maria PaolaBataille, ArnaudMay, StephenBeechler, BriannaMcCauley, JohnBell, MelindaMcConkey, SandraBenazzi, CinziaMcGiven, JohnBennett, PeterMcNulty, Margaret A.Bentley, EllisonMeade, Kieran G.Bernstein, MarkMeehan, Michael P.Beths, ThierryMellies, JayBickerton, EricaMena, IgnacioBidarimath, MallikarjunMendonca, AubreyBlack, William CMenezes, Irwin Rose AlencarBobe, GerdMentaberre, GregorioBöhmer, ChristineMichel, PiotrBongiovanni, LauraMlera, LuwanikaBoqvist, SofiaMolento, CarlaBorzacchiello, GiuseppeMoore, Phillip AnthonyBown, KevinMoore, SusanBrown, MarkMorton, DavidBrowning, Glenn FMosedale, PamBrugger, KatharinaMuckle, Catherine AnneBulow, LeifMüller, Ralf S.Buonavoglia, DomenicoMutinelli, FrancoBurges, GrahamNaguib, MahmoudCaires, KyleNarute, PurushottamCalero-Bernal, RafaelNelson, LauraCambier, CaroleNickbakhsh, SemaCarducci, Bernardo J.Nokireki, TiinaCésar Cristiano, BassettoNorman, Elizabeth J.Chang, Geng-RueiNüesch-Inderbinen, MagdalenaChen, KangmingNuttall, Patricia AChen, Hui-WenOgden, Nicholas HumeChen, ZhihangOkumura, HirokiChruszcz, MaksymilianOppliger, AnneCorah, LouisePaixao Coelho, Jose AugustoCorrea, MaríaPali-Schöll, IsabellaCribb, Alastair E.Pantin-Jackwood, MaryDame, John B.Parés-Casanova, Pere M.Davis, Michael E.Pas, SuzanDavis, ElizabethPasick, JohnDe Rooster, HildePasicka, EdytaDec, MartaPeralta, SantiagoDell, ColleenPérez, ValentinDellamaria, DeboraPérez-Sánchez, RicardoDervisis, NickPetrovski, KiroDimitrios, VagenasPhalen, DavidDkhar, Hedwin KitdorlangPietrantonio, Patricia VictoriaDomingues, SaraPinnapireddy, Shashank ReddyDorman, David C.Pirrone, FedericaDrögemüller, CordPoli, AlessandroDunshea, FrankPoli, AlessandroEconomou, VangelisPushko, PeterEl-Gazzar, Mohamed M.Quessy, SylvainElmi, AlbertoRahe, MikeEschbaumer, MichaelRawlings, John MerrittFagerholm, VeronicaRehbein, SteffenFaleiro, Maria L.Reichard, MasonFan, ZhijinReif, Kathryn E.Fawcett, AnneReifinger, MartinFei, Andrew Chang-YoungReiter, AlexanderFERRO, SilviaReuter, TimFlamini, GuidoRhodes, GlennForget, PatriceRhyan, JackFyfe, JohnRiccardo, OrusaGaardbo Kuhn, KatrinRocca, StefanoGalisteo Jr., Andres JimenezRodriguez Valle, ManuelGama, AdelinaRose, HannahGarikipati, DilipRyser, ElliotGaunt, EleanorSalak-Johnson, JaneenGazzano, AngeloSalb, AmandaGillett, JamesSambo, MagangaGilot-Fromont, EmmanuelleSang, YongmingGioia, GloriaSant’Ana, Manuel MagalhãesGiudice, ChiaraSantos-Gomes, GabrielaGiuliano, AntonioSanz, IvanGodfroid, JacquesSase, AjinkyaGogal, Robert M.Sassera, DavideGómez, José B. RodriguezSaxena, VikasGonzalez-Reiche, Ana SilviaSchweigert, FlorianGradinaru, Andrei CristianScott, IanGramiccia, MarinaSerrano-Perez, BeatrizGranger, L. AbbigailSharafeldin, Tamer A.Granier, SophieShaw, Dana K.Greer, AndyShaw, DanaGrosche, AstridShek-Vugrovečki, AnaGulia-Nuss, MonikaShen, ZhenyuGurramkonda, ChandrasekharShiff, CliveHabib, IhabShil, NirajHahn, CarolineShivley, Jacob M.Hamilton, James P.Simitzis, PanayiotisHammerl, Jens AndreSingh, PallaviHan, MingyuanSkorupski, KatherineHassan, Sharifah SyedSlater, MargaretHazel, SusanSmith, GrahamHeller, MartinSolounias, NikosHelmy, Yosra A.Sondgeroth, KerryHerrtage, MichaelSoultos, Nikolaos D.Herzog, HaroldSparger, Ellen (Liz)Hess, AngelaSpiegler, VerenaHickling, Graham J.Sponseller, BrettHielm-Björkman, AnnaStanley, Scott D.Hildreth, BlakeStear, MichaelHirai, ItaruSteinman, AmirHoff, RodneyStrandberg, ErlingHolzel, ChristinaStromberg, ZacharyHuijbers, PatriciaTabor, AlaHulsebosch, SeanTajima, AtsushiIlha, MarciaTava, AldoJacques, BarratTellez, GuillermoJahns, HanneTerrazzano, GiuseppeJarvis, JodieTheuns, SebastiaanJayamohan, HarikrishnanThiry, DamienJordan, KieranThomas, Donald B.Kajamies, AnuTomaso, HerbertKale, AbhijitTorsteinsdottir, SigurbjorgKarim, ShahidTu, ChangchunKawashima, ChihoUmhang, GéraldKawazu, Shin-ichiroUpadhyay, AbhinavKeane, Orla M.Vainio, OutiKedrowicz, April AnnValcárcel Sancho, FélixKetzis, Jennifer K.Van Wettere, ArnaudKim, Shin-HeeVarcasia, AntonioKlompen, HansVentura Queija, JacintKnobel, DarrynVicente, JoaquínKol, AmirVrentas, Catherine E.Kong, WeiliWaal, Theo DeKook, Peter HendrikWales, AndrewKöster, WolfgangWallace, RyanKurtti, TimothyWang, Ching-HoLadevèze, SandrineWells, James E.Le Goffic, RonanWeston, Jennifer F.Lechenne, MoniqueWhite, Amelia G.Leeb, TossoWhiting, TerryLennox, Angela M.Whittaker, AlexandraLerner, HenrikWoltenberg, LeslieLevingstone, Tanya J.Wu, HongzhuanLi, Jiao JiaoYool, DonaldLi, ShitaoZaoutis, TheoklisLindahl, JohannaZarfel, Gernot E.Löhr, Christiane V.Zmora, PawelLudvigsen, JaneZohari, SiamakLuedtke, Brandon


